# Could Vaspin Be a Potential Diagnostic Marker in Endometrial Cancer?

**DOI:** 10.3390/ijerph20064999

**Published:** 2023-03-12

**Authors:** Dominika Pietrzyk, Piotr Tkacz, Mateusz Kozłowski, Sebastian Kwiatkowski, Małgorzata Rychlicka, Ewa Pius-Sadowska, Bogusław Machaliński, Aneta Cymbaluk-Płoska

**Affiliations:** 1 Department of Reconstructive Surgery and Gynecological Oncology, Pomeranian Medical University in Szczecin, Al. Powstańców Wielkopolskich 72, 70-111 Szczecin, Poland; 2 Department of Obstetrics and Gynecology, Pomeranian Medical University in Szczecin, Al. Powstańców Wielkopolskich 72, 70-111 Szczecin, Poland; 3 Department of Nursing, Pomeranian Medical University, 71-210 Szczecin, Poland; 4 Department of General Pathology, Pomeranian Medical University in Szczecin, Al. Powstańców Wielkopolskich 72, 70-111 Szczecin, Poland

**Keywords:** vaspin, cancer, endometrial cancer, diagnostic, diagnostic factor, obesity, insulin resistance

## Abstract

Obesity and being overweight are risk factors for many types of cancer, including endometrial cancer. Adipose tissue is thought to be an endocrine organ that produces various hormones, including one known as vaspin. Insulin resistance, metabolic syndrome and type 2 diabetes are all associated with higher vaspin levels. A total of 127 patients divided into study (endometrial cancer) and control groups (non-cancerous) participated in this research. Serum vaspin levels were measured for all patients. The analysis was performed while taking into account grading and staging. In order to assess the usefulness of the tested protein as a new diagnostic marker, we used the plotting of a curve (ROC) and the calculation of the AUC curve to characterize the sensitivity and specificity of the parameters tested. We concluded that there were significantly lower vaspin levels in patients with endometrial cancer compared to patients with benign endometrial lesions. Vaspin may be a useful diagnostic marker in separating benign lesions from endometrial cancer.

## 1. Introduction

Endometrial cancer (EC) is the sixth most common malignancy for women worldwide [1]. Its incidence is increasing, particularly in postmenopausal women. Only 4% of patients are under the age of 40 [2]. More than half of endometrial cancer cases are attributed to obesity, which has been identified as an independent risk factor for the disease [3]. Research confirms the link between obesity, hyperinsulinemia, type 2 diabetes and endometrial cancer [4,5,6,7]. The increase in cellular reactivity to insulin is connected with the activation of the MAPK/PI3K/AKT/mTOR signaling pathway, which is specific to cancer (EC) [8]. Often, this pathomechanism is further enhanced by the loss of the PTEN suppressor gene, which normally acts in opposition to the PI3K/AKT/mTOR pathway [9]. Bioavailable estrogens, especially when unopposed by progesterone, may increase the risk of EC through mitogenic effects in endometrial tissue [10]. Type I endometrial tumors usually express high levels of estrogen receptor (ER), and they are thought to be hormonally driven as opposed to type II endometrial cancer [11]. A low level of SHBG (sex hormone binding globulin) is induced by high body weight. Observations suggest a negative correlation between circulating SHBG levels and insulin resistance (IR). Decreased SHBG levels increase the bioavailability of androgens, which in turn leads to the progression of ovarian pathologies such as, among others, polycystic ovarian syndrome (PCOS) [12]. In addition, the effect of hormonal pathomechanisms can be a state of hyperprolactinemia, which further stimulates adrenal androgen production. Similarly, in the course of negative feedback in hypothyroidism, there is a stimulation of TSH (thyroid-stimulating hormone), which is also responsible for the state of hyperprolactinemia and affects proper sex hormone management [13,14]. As a result, the patient develops a state of relative hyperestrogenism. Therefore, obesity correlates with an increased risk of endometrial hyperplasia and, ultimately, EC [15]. Adipose tissue is seen as an endocrine organ, synthesizing so-called adipocytokines such as, among others, vaspin, which belongs to the serine protease inhibitor family [16,17]. After menopause, adipose tissue becomes the main location of estrogen synthesis and the source of aromatase, the enzyme responsible for converting androgens to estrogens. After binding to their receptors, estrogens can indirectly affect the transcription of such known proliferative factors as IGF1R and IGF1 [18]. It acts directly by stimulating endometrial proliferation through the MAPK and AKT signaling pathways. The use of single-ingredient hormonal contraception and hormone replacement therapy based solely on estradiol, although rarely practiced these days, increases the risk of the aforementioned pathomechanisms. A drug that can increase proliferation and, thereby, the risk of abnormal lesions is tamoxifen, which is used in the treatment of breast cancer [19]. It is well-established that a state of hyperestrogenism unbalanced by progesterone contributes to a significantly higher risk of endometrial cancer type 1 and its precursors [8,20]. By understanding the mechanisms of estrogens and progestogens in the endometrium, their undeniable proliferative and antiproliferative effects can be noted.

Vaspin is an adipokine found in many tissues. Unlike the vast majority of cytokines, it has anti-inflammatory and antiproliferative effects by inhibiting inflammatory mediators such as NF-κB. Moreover, vaspin inhibits insulin degradation, thereby improving glucose tolerance. At the same time, it also has the ability to inhibit IRS-2 (Insulin Receptor Substrate 2) phosphorylation, a protective mechanism against the onset of tissue hyperinsulinemia [21]. Increased vaspin expression is observed in patients with type II diabetes, obesity and metabolic syndrome [7]. As low-grade inflammation and insulin resistance play an important role in the pathogenesis of endometrial cancer, we wondered whether there is a link between the occurrence of endometrial cancer and vaspin concentrations and if it is possible to use vaspin as a diagnostic marker in endometrial cancer. Despite ongoing research, no useful marker for endometrial cancer has yet been found. The purpose of this study was to determine the utility of vaspin in the diagnosis of endometrial cancer. In addition, we also investigated whether vaspin is useful in distinguishing grades and stages of endometrial cancer.

## 2. Materials and Methods

### 2.1. Participation in the Study

A total of 127 patients with abnormal uterine bleeding/abnormal ultrasound images from the Department of Gynecological Surgery and Gynecological Oncology at the Pomeranian Medical University in Szczecin, Poland were included in the study. Lack of patient consent, endometrial hyperplasia diagnosed histopathologically, acute inflammation, other cancers, collagenosis, chronic kidney disease, cirrhosis, therapy with biological agents and immunotherapy were among the exclusion criteria. The material for the study was collected over a period of 24 months. The Pomeranian Medical University’s Ethical Committee approved the study (approvement no. (KB-0012/148/2020), and each participating patient signed an informed consent form in order to take part.

### 2.2. Classification of Patients into Study and Control Groups

Patients were divided into two groups according to the histological diagnosis obtained by endometrial biopsy, curettage or hysteroscopy. Group A, consisting of 62 patients who had benign endometrium lesions, was separated into two subgroups: A1 (endometrial polyps, *n* = 30) and A2 (uterine myomas, *n* = 32). Group B included 65 endometrial cancer patients. The group is described in Table 1.

### 2.3. Preparation of Pre-Laboratory Samples

All patients’ serum vaspin levels were assessed before surgical treatment. Following surgical intervention in the study group, analysis was carried out while accounting for the tumor’s histopathological differentiation (grading) and clinical stage (staging). The patients were split into two groups following a histopathological examination. The characteristics of the group, taking into account grading and staging, are shown in Table 2.

### 2.4. Laboratory Analysis

Following centrifugation of the blood samples and freezing of the resultant serum in Eppendorf-style containers maintained at −80 °C, biochemical analyses were carried out. Vaspin concentrations were measured in serum by using an immunoenzymatic ELISA-multiplex fluorescence assay (Luminex Corporation, Austin, TX, USA) and utilizing a commercial Bio Plex Pro RBM Human Metabolic Panel 2 (Biorad, Hercules, CA, USA).

### 2.5. Statistical Analysis

The statistical evaluations were performed using Statistica version 10 PL software. The Shapiro–Wilk test was used to determine whether the study’s variables have a normal distribution. With the exception of the population-wide variable age, which has a normal distribution, none of the other variables have normal distributions. As a result, non-parametric methods (Spearman’s rank correlation coefficient) and non-parametric significance tests were used in the analysis to test their relationships. The assumption that the distributions of the two variables are representative of the same populations was confirmed using the Mann–Whitney U test of significance for independent samples. A non-parametric Kruskal–Wallis significance test was used to corroborate the hypothesis whether samples originated from the same distribution. The populations that varied were examined using post hoc tests. We used the plotting of a curve (ROC) and the calculation of the area under the curve (AUC) to characterize the sensitivity and specificity of the parameters tested in order to determine the usefulness of the tested protein as a new diagnostic marker. A value of *p* < 0.05 was considered an indicator of statistical significance.

## 3. Results

### 3.1. Characteristics of the Study Group

There were statistically significant differences between the rates of pre- and postmenopausal women in groups of patients with endometrial cancer and benign lesions. The group of patients with diagnosed EC included 13 premenopausal and 52 postmenopausal patients, whereas the control group contained 27 premenopausal and 35 postmenopausal patients. In addition, we divided the groups according to the presence of endometrial cancer risk factors such as hypertension, body mass and type II diabetes. The smallest group of EC patients was the normal-weight group, which was nearly equal in population to the overweight patient group (*p* = 0.031). Statistically significant differences were found among female patients with benign changes with BMI 18.9–24.9 (normal weight) compared to BMI 25–29.9 (pre-obesity). In the study group, the numbers of pre-obese and normal-weight patients were 25 and 14, respectively. In the control group, there were 24 pre-obese and 22 normal-weight patients. The results are described in Table 3.

### 3.2. Evaluation of Serum Vaspin Levels in Relation to Histopathological Diagnosis

**Figure 1:**
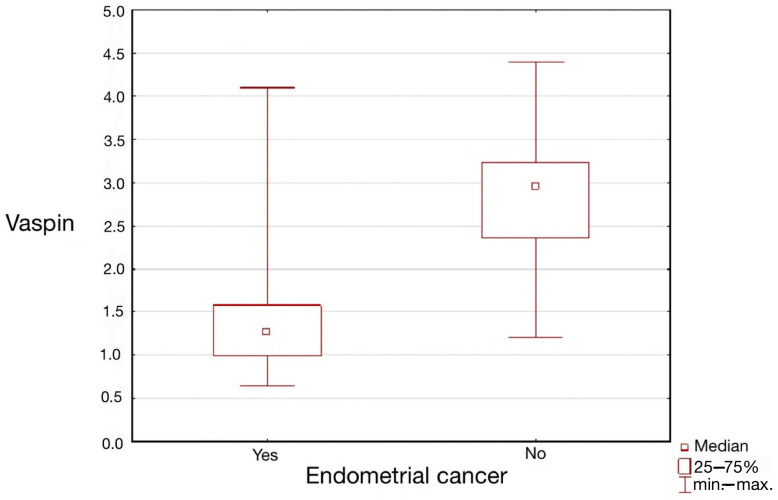
Graphical representation of median vaspin concentrations in the study and control groups.

Median vaspin concentrations were significantly lower in patients with endometrial cancer in the study group compared to the median vaspin serum concentrations in the control group (*p* = 0.001). Statistically significant differences were observed in patients with EC compared to patients with endometrial polyps (*p* = 0.016) and uterine myomas (*p* = 0.028). However, statistically significant differences were not revealed between median concentrations of serum vaspin in the group of patients with endometrial polyps vs. patients with uterine myomas. The results are presented in Table 4 and Figure 1.

### 3.3. Assessing the Relationships of Serum Vaspin Levels in Patients with Endometrial Cancer at the Time of Collection

Statistically significant differences were not revealed between the median concentration of vaspin in the group of patients at the time of diagnosis and 6–8 weeks later before surgical treatment. The results are presented in Table 5.

### 3.4. Evaluation of Vaspin as a New Diagnostic Marker— ROC Curve Analysis for Vaspin Protein Relative to Study and Control Group

**Figure 2:**
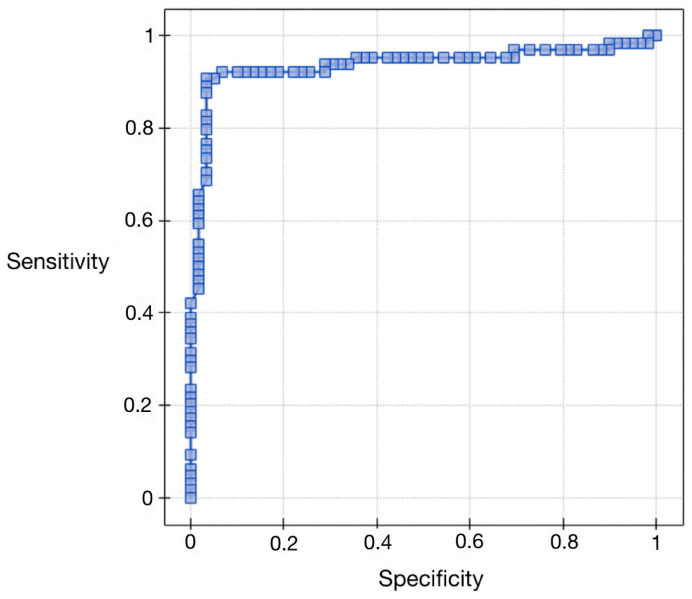
ROC curve for serum vaspin concentrations relative to the study and control groups.

In order to evaluate the diagnostic values of vaspin, ROC curves were plotted, and the areas under the ROC curves (AUC) were calculated. For patients with endometrial cancer and benign endometrial lesions, the AUC was 0.88 (see Figure 2 and Table 6 below). The conclusion can be drawn that checking serum levels of vaspin before surgery can be a good diagnostic test to differentiate benign lesions from endometrial cancers. The data obtained from the appearance of ROC curves according to the hormonal status of patients were different. The AUC for premenopausal patients was 0.76, and for postmenopausal patients, it was 0.92.

### 3.5. Evaluation of Vaspin Protein as a Differential Test Relative to Grading—ROC Curve Analysis for Vaspin Protein Compared to Histopathological Differentiation

**Figure 3:**
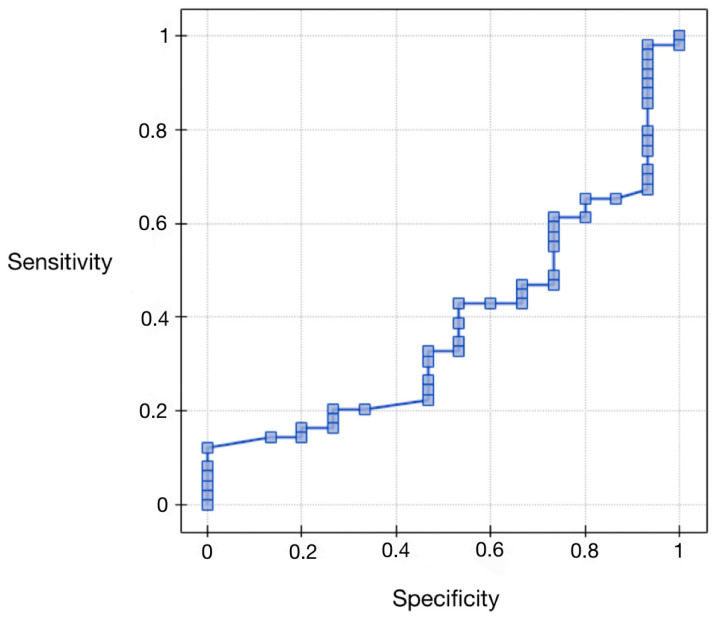
ROC curve for serum vaspin concentrations compared to histopathological differentiation.

Regarding the area under the AUC curve (0.38), we found that it was less than 0.5. Therefore, preoperative serum concentrations of vaspin cannot be considered for diagnostic use in the differential grading of endometrial cancer. The results are presented in Figure 3 and Table 7.

### 3.6. Evaluation of Vaspin Protein as a Differential Test Relative to Staging—ROC Curve Analysis for Vaspin Protein Depending on the Clinical Stage

**Figure 4:**
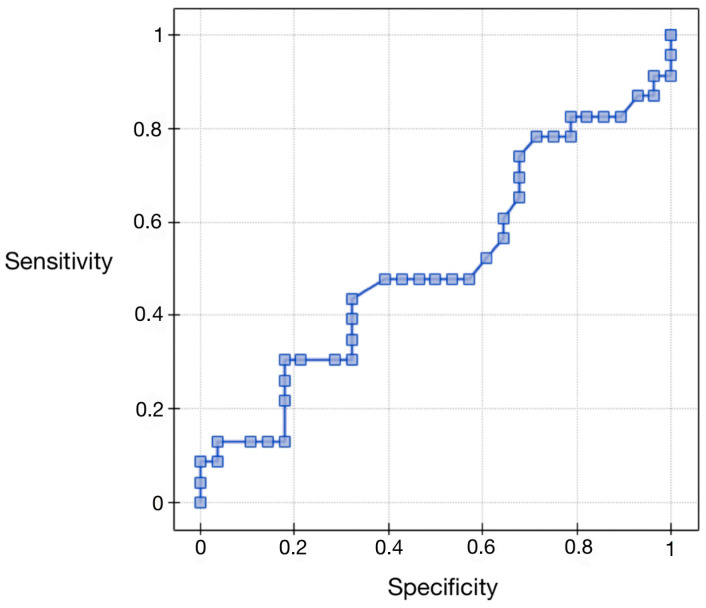
ROC curve for serum vaspin concentrations depending on the clinical stage.

Regarding the area under the AUC curve (0.51), we found that it was greater than 0.5. Therefore, preoperative serum concentrations of vaspin can be considered for diagnostic use in the differential staging of endometrial cancer. The results are presented in Figure 4 and Table 8.

### 3.7. Evaluation of the Sensitivity and Specificity of Vaspin as a Diagnostic Factor in Endometrial Cancer

Table 9 presents the percentages of sensitivity and specificity for vaspin as a diagnostic factor in the entire study group of patients, with distinction between the subgroups of premenopausal and menopausal patients. We found that sensitivity and specificity in the entire group were 86% and 78%, respectively. For premenopausal patients, the results were 81% sensitivity and 66% specificity, and they were 88% sensitivity and 72% specificity for menopausal patients.

## 4. Discussion

Overweight or obese people represent 60% of the European population [22]. Obesity predisposes people to a number of metabolic disorders, such as insulin resistance, type 2 diabetes, hypertension and dyslipidemias. It is also a significant risk factor for cardiovascular disease and a number of cancers, particularly endometrial, ovarian, breast, pancreatic and colorectal cancers [23]. In our study, 61.9% of patients with endometrial cancer were noted to be obese. In the control group, the percentage of obese patients was much lower at 38.1%. However, the increase in these risks is not linearly related to weight gain [24]. The presence of hyperestrogenism, inflammation and insulin resistance due to obesity with the associated metabolic syndromes increases the risk of oncogenesis [25]. Free fatty acids (FFAs), which are increased in obese individuals, stimulate TLR4 receptors, inducing the expression of metabolic pathways that promote inflammation [26]. Furthermore, studies show that inflammatory factors may induce IR (insulin resistance) through the NF-κB interaction [27]. A meta-analysis conducted by the Agency for Research on Cancer (IARC) in 2016 clearly found that the risk of endometrial cancer increases with BMI. The patients’ BMI-dependent relative risks were approximately 1.5 for pre-obese patients (BMI 25–29.9) compared to patients with diagnosed class III obesity (BMI > 40), for whom the relative risk (RR) was 6.25 [28].

Adipokines are polypeptide cytokines produced by the adipose tissue, and they are especially important in obesity-related cancers. Most of them have pro-inflammatory properties and are increased in cancers [29]. However, Li et al., point to the anti-inflammatory properties of one of the adipokines, called vaspin [30]. In our study, we focused our attention on vaspin, a part of the serpin (serine protease inhibitor) family, also called serpin A12 [31]. Pich et al. in a 2021 study reported its presence in many glands, such as the hypothalamus, pancreas, thyroid gland, ovaries, placenta and testes. Vaspin levels were proven to be elevated in type 2 diabetes, metabolic syndrome, obesity, coronary artery disease and insulin resistance [32,33]. In addition, it blocks the activation of NF-κB in endothelial and pancreatic cells, preventing the development of inflammation. By inhibiting Kallikrein 7, vaspin blocks insulin degradation, resulting in reduced insulin resistance and improved glucose tolerance [34]. In our study, we observed statistically significant differences in serum levels between patients with BMI levels indicating pre-obesity or obesity. As mentioned previously, insulin resistance and chronic low-grade inflammation induced by obesity are important risk factors for endometrial cancer. The multiple beneficial effects of vaspin as a factor in minimizing these conditions led us to investigate its use as a diagnostic marker for endometrial malignancies.

The incidence of hypothyroidism in patients with endometrial cancer is significantly elevated, and pretreatment serum TSH (thyroid-stimulating hormone) levels are an independent risk factor for EC. Based on a review of available experimental and clinical data, hypothyroidism is closely associated with many EC risk factors, including metabolic syndrome, PCOS and infertility [35]. Seebacher et al. concluded that TSH levels were correlated with dyslipidemia [36,37]. Considering carbohydrate metabolism, our study shows that the concentrations of serum vaspin were higher in patients with diabetes, but the result was not statistically significant. Although the effect of vaspin in reducing insulin resistance is known, there are few reports that relate serum concentrations of vaspin in patients with heart disease, including those with hypertension. In our study, we found no differences in vaspin concentrations according to the presence of arterial hypertension. Other studies showed that vaspin can inhibit vascular endothelial apoptosis and cause weakness in blood vessels stimulated by high glucose levels [38,39]. Körner et al. achieved results different from our study. They demonstrated that serum vaspin levels correlated with blood pressure and may be associated with vascular endothelial damage [40].

In contrast to patients with benign endometrial lesions, patients with endometrial cancer have considerably decreased vaspin levels, according to our study. Significant reductions of vaspin levels in patients with endometrial cancer relative to patients with benign lesions were also found in earlier studies [21,41]. We found no statistically significant differences in vaspin concentrations between patients with endometrial polyps and uterine myomas. We also examined the dependence of vaspin concentrations on the time of sampling. There were no statistically significant differences in vaspin concentrations taken at the beginning of the diagnostic process and 6–8 weeks later before surgical treatment. To investigate the usefulness of vaspin as a new preoperative diagnostic marker, we used the ROC curve and calculated the AUC, which was 0.88. We also evaluated the use of vaspin as a differentiating factor for grading and staging endometrial cancer, and we obtained AUCs of 0.38 and 0.51, respectively.

There is a connection between pathologies linked to hormonal imbalance and significantly increased vaspin in obese patients. Insulin resistance was directly correlated with vaspin mRNA expression but not with its circulating levels [42]. As a result, we speculated that elevated vaspin levels could be a compensatory mechanism in the beginnings of the early stages of insulin resistance. In addition, Tan et al. found that vaspin synthesis is stimulated by glucose in omental adipocytes [43]. According to epidemiological and clinical evidence, the development of EC is significantly influenced by insulin resistance and the accompanying hyperinsulinemia. It was also proven that the risk of developing EC rises quite quickly after the diagnosis of IR and diabetes, or nearly 6 months after their detection [44]. The effects of vaspin also extend to other physiological processes, including food intake and inflammation [45]. Undoubtedly, obesity is a significant factor that contributes to the progress of endometrial cancer [46].

Vaspin appears to be a promising indicator protein that can be used in patients with abnormal bleeding. More research on vaspin as a diagnostic factor in endometrial cancer remains to be considered.

## 5. Conclusions

Vaspin may be a potential diagnostic marker to be used to differentiate endometrial cancer from benign lesions. It is not possible to distinguish grades of endometrial cancer using the tissue expression of vaspin. However, vaspin can be useful for distinguishing clinical stages of endometrial cancer.

## Figures and Tables

**Table 1:** Division of patients according to risk factors for endometrial cancer: BMI, body mass index; HA, arterial hypertension; DM type 2, diabetes mellitus type 2.

Characteristics		Patients, *n* (%)
BMI [kg/m^2^]	18.5–24.9	14 (22%)
	25–29.9	25 (38%)
	≥30	26 (40%)
HA	no	24 (37%)
	yes	41 (63%)
DM type 2	yes	40 (62%)
	no	25 (38%)
Hormonal state	premenopausal state	13 (20%)
	postmenopausal state	52 (80%)
Hormone replacement therapy	yes	37 (57%)
	no	28 (43%)

**Table 2:** Division of endometrial cancer patients: G1–3, grading 1–3; FIGO, International Federation of Gynecology and Obstetrics.

Groups	Distribution	Patients, *n* (%)
Histopathological grade of the tumor	G1	18 (28%)
	G2	32 (49%)
	G3	15 (23%)
Clinical stage of the tumor	FIGO I and II	46 (71%)
	FIGO III and IV	19 (29%)
Lymph node metastases	Yes	19 (29%)
	No	46 (71%)
LVSI (Lymphovascular space invasion) metastases	Yes	39 (60%)
	No	26 (40%)
Presence of angioinvasion	Yes	30 (46%)
	No	35 (54%)

**Table 3:** Characteristics of female patients: BMI, body mass index; HA, arterial hypertension; DM type 2, diabetes mellitus type 2.

Diagnosis	Premenopausal State (PM)				Postmenopausal State (M)					*p*
	*n*			%	*n*	%				
Endometrial cancer	13			32.5	52	59.8				0.031
Non-EC	27			67.5	35	40.2				0.027
	HA—yes				HA—no					*p*
	*n*			%	*n*	%				
Endometrial cancer	41			60.3	24	40.7				0.033
Non-EC	27			39.7	35	59.3				0.008
	DM type 2—yes				DM type 2—no					*p*
	*n*			%	*n*	%				
Endometrial cancer	40			51.4	25	51.0				NS
Non-EC	38			48.7	24	49.0				NS
	BMI 18.9–24.9		BMI 25–29.9		*p*	BMI ≥ 30		BMI 25–29.9		*p*
	*n*	%	*n*	%		*n*	%	*n*	%	
Endometrial cancer	14	38.9	25	51.0	0.031	26	61.9	25	51.0	NS
Non-EC	22	61.1	24	49.0	0.042	16	38.1	24	49.0	0.049

**Table 4:** Vaspin concentrations in the endometrial cancer group and in the control group, including subgroups of the control group.

Statistical Parameters	Vaspin (ng/mL)		*p*
	Endometrial Cancer	Control Group	
Range	0.3–2.9	0.7–6.6	0.001
Median	1.3	3.1	
Confidence interval	0.8–1.9	2.8–3.8	
Standard deviation	0.1	0.3	
	Vaspin (ng/mL)		*p*
	Endometrial cancer	Endometrial polyps	
Range	0.3–2.9	0.9–6.2	0.016
Median	1.3	5.1	
Confidence interval	0.8–1.9	4.6–6.1	
Standard deviation	0.1	0.7	
	Vaspin (ng/mL)		*p*
	Endometrial cancer	Uterine myomas	
Range	0.3–2.9	0.8–5.9	0.028
Median	1.3	3.3	
Confidence interval	0.8–1.9	3.2–5.2	
Standard deviation	0.1	0.6	
	Vaspin (ng/mL)		*p*
	Uterine myomas	Endometrial polyps	
Range	0.8–5.9	0.9–6.2	NS
Median	3.3	5.1	
Confidence interval	3.2–5.2	4.6–6.1	
Standard deviation	0.6	0.7	

**Table 5:** Serum vaspin concentrations in patients before diagnostic sampling and before surgical treatment.

Statistical Parameters	Vaspin (ng/mL)		*p*
	Endometrial Biopsy, Curettage of the Uterine Cavity, Hysteroscopy	Before Surgical Treatment	
Range	0.2–4.1	0.1–3.8	NS
Median	1.8	1.2	
Confidence interval	1.2–2.0	0.7–1.6	
Standard deviation	0.2	0.1	

**Table 6:** ROC curve analysis for serum vaspin concentrations relative to the study and control groups.

**SE (AUC)**	0.025641
**−95% Cl**	0.886715
**+95% Cl**	0.987226
***p*** **-value**	<0.000001

**Table 7:** ROC curve analysis for serum vaspin concentrations compared to histopathological differentiation.

**AUC**	0.384354
**SE (AUC)**	0.080177
**−95% Cl**	0.22721
**+95% Cl**	0.541498
**Statistics Z**	−1.347129
***p*** **-value**	0.177939

**Table 8:** ROC curve analysis for serum vaspin concentrations depending on the clinical stage.

**AUC**	0.50854
**SE (AUC)**	0.084717
**−95% Cl**	0.342498
**+95% Cl**	0.674583
**Statistics Z**	0.104114
***p*** **-value**	0.917079

**Table 9:** Evaluation of the sensitivity and specificity of vaspin in relation to the hormonal status of patients: PM, premenopausal state; M, menopausal state.

	Sensitivity	Specificity
Vaspin (entire group)	86%	78%
Vaspin (PM subgroup)	81%	66%
Vaspin (M subgroup)	88%	72%

## Data Availability

The data presented in this study are available from the corresponding author, D.P., upon reasonable request.
